# Facelifts and Professional Success: Can a Facelift Increase Competitive Edge and Save a Patient's Job?

**DOI:** 10.1093/asj/sjae174

**Published:** 2024-07-31

**Authors:** Foad Nahai, Michelle McGill

“Thank you, Dr Nahai, the facelift saved my job, gave me another 10 years. I do not look old anymore and will not be pushed into retirement!”—Helen Cleveland, personal communication, June 2024.

Are we as aesthetic surgeons able to “save jobs” for our patients? Helen is not the only one of our patients who confirms we can—and we do. Over the years, both male and female patients have said to the senior author (F.N.) that they are seeking facial rejuvenation to help them compete with younger individuals for jobs and promotions in the workplace. This has been especially true for those in careers that entail working face-to-face with clients, in which a youthful appearance may be perceived as having energy and vitality. These careers have included real estate agents, salespersons, and even physicians who felt that their facial appearance and perception of being “old” made them less competitive. Although ageism in the workplace is frowned upon and illegal in some countries, including the United States, it is undeniable that younger-looking individuals have an advantage. One can argue that an older individual brings more experience and, dare I say, judgment; however, an old- or tired-looking face may detract from those attributes.

Obviously, a facelift or any cosmetic procedure does not improve an individual's skills, knowledge, or experience—all of which are typically considered in hiring decisions. Although that may be true, it is undeniable that, given the choice, most would choose to do business with someone perceived as younger or more attractive.^1^ Our purpose here is not to discuss whether this is right or even ethical but merely to point out the fact and discuss how cosmetic procedures and aesthetic surgery can impact career opportunities and “save jobs.” In some professions, such as the entertainment industry, TV anchors and announcers with a youthful and attractive appearance may have an advantage. We are confident that countless colleagues have taken care of individuals in these professions, as has F.N., so that those in the public eye can remain employed.

We have mentioned beauty and attractiveness above as if they are the same. They, of course, are not. Whereas beauty may refer to facial appearance or body form, attractiveness goes beyond just looks. It includes many nonvisual qualities such as a warm personality, likability, confidence, and a positive outlook, often referred to as “inner beauty.” Aesthetic surgery boosts self-esteem, confidence, and feeling good about how one looks, which often leads to more confidence and improved job performance. Although there is strong evidence that those who are more attractive are more likely to be hired and earn more, attractiveness and youth are not the same.^2^ However, there is an overlap: looking younger may be perceived as more attractive and energetic, which is where we as aesthetic surgeons can play a role in enabling our patients to remain employed and to continue to be productive. Bashour links youthfulness with attractiveness, listing the 4 most important determinants of attractiveness as averageness, sexual dimorphism, youthfulness, and symmetry.^3^ Mathes and coauthors explore the relationship between aging and attractiveness, reporting a negative correlation between increasing age and attractiveness.^4^ Henns's experiment with photographs confirms the negative correlation between perceived age and attractiveness in females, but, interestingly, not in males.^5^ Jones studies the relationship between neoteny and sexual selection and attractiveness, concluding that a more youthful appearance has a positive correlation with attractiveness.^6^ An online survey conducted by Maestripieri finds a negative correlation between age and attractiveness.^7^ We can safely conclude that there is indeed a relationship between attractiveness and age.

There is ample evidence that the psychological effects of aging include a loss of self-esteem and diminishing of social power, sexual identity, and position in the workplace.^8-10^ Aesthetic surgery boosts self-esteem, and feeling good about how one looks can naturally lead to more confidence and improved job performance. Spector reports on a Harvard study in which females with makeup are regarded by others not only as better looking but more likable, competent, and trustworthy.^11^ He also cites a Dutch study in which attractive managers are more successful in their dealings with clients. A University of Wisconsin study of S&P 500 companies reports higher revenues in companies with attractive CEOs.^12^ A Finnish study on incumbent political candidates notes that the more attractive individuals on average scored 20% more votes.^13^ The study suggests that voters enjoy watching the better-looking individuals, and that in politics attractiveness is an advantage.

Finally, attractiveness and beauty rely on the feelings they instill in oneself and others. The self-esteem and confidence enhanced or regained following aesthetic surgery restores in oneself the feeling of being younger, and also gives others the impression of vitality and youth. Helen relates to me that after her facelift she felt a boost in her confidence and was smiling more often, which was also noted by her coworkers. Through aesthetic surgery and cosmetic treatments, we can and do enable our patients to increase their longevity and competitive edge in the workplace.

Yes, facelifts can save jobs!
